# Hodgkin Lymphoma at the Paediatric Oncology Unit of Gabriel Touré Teaching Hospital, Bamako, Mali: 5-Year Experience

**DOI:** 10.1155/2011/327237

**Published:** 2011-02-10

**Authors:** B. Togo, F. Traoré, A. P. Togo, C. B. Traoré, K. Dumke, M. Diawara, A. A. Diakité, M. Sylla, F. Traoré-Dicko, B. Traoré, T. Sidibé

**Affiliations:** ^1^Service of Pediatrics Gabriel Touré Teaching Hospital, Bamako BP 267, Mali; ^2^Surgery Service Gabriel Touré Teaching Hospital, Bamako BP 267, Mali; ^3^Service of Pathology INRSP, Bamako BP 267, Mali; ^4^Department of Pathology, University of Münster, 48749 Münster, Germany

## Abstract

*Introduction*. The aim of this retrospective, unicentric study over 5 years is to describe the epidemiologic, pathologic, clinic and therapeutic aspects of children treated for Hodgkin lymphoma in our paediatric oncology unit. *Patients and Methods*. From January 2005 to December 2009, all children under 18 years of age, with Hodgkin lymphoma were included in this study. The treatment protocol was the GFAOP (Groupe Franco—Africain d'Oncologie Pédiatrique) Hodgkin lymphoma treatment protocol. *Results*. During the study period, 217 cancer cases were diagnosed in our centre. Of these cases, 7 were Hodgkin Lymphoma (LH) (0.04%). The mean age was 11.7 years. The sex-ratio was 6/1. 4% (5/7) of patients were stage IIB and 28.6% (2/7) stage IIIB of Ann-Arbor classification. There were 3 cases (42.8%) of sclero-nodular subtype, 2 cases (28.6%) of lymphocyte-rich classical HL subtype, 1 case (14.3%) of mixed cellularity and 1 case (14.3%) of lymphocyte depleted subtype. With a median followup of 37 months, 5 patients (71.4%) are alive, and 2 patients (28.6%) died. *Conclusion*. Broader multicentric studies are needed for more accurate data on this malignancy.

## 1. Introduction

Hodgkin's disease, today known as Hodgkin lymphoma, is a malignant tumor of the lymphoid tissue which accounts for less than 1% of all new cases of cancer throughout the world [1, 2]. 

About 20 000 new cases of Hodgkin lymphoma are diagnosed each year in North America and Europe [3].

 While the annual incidence of non-Hodgkin lymphoma is 2.2 to 3.8 cases per 100 000 inhabitants in Central Africa, that of Hodgkin lymphoma is unknown in most parts of Africa [4]. 

While publications on the disease from Western countries and the rest of the world are numerous in medical literature, very few data are available in Africa [5].

The aim of this retrospective study over five years (median followup of 37 months) is to describe the epidemiological, pathological, clinical, and therapeutic aspects of all the cases of Hodgkin lymphoma treated in our pediatric oncology unit of Gabriel Touré Teaching Hospital in Bamako. 

## 2. Patients and Methods

Mali is one of the most underprivileged countries in the world [6]. Socioeconomic problems limit access to health care for most citizens and consequently, the treated cases do not reflect the actual epidemiology of the disease.

Our pediatric oncology unit is the only structure in the country specialized in the treatment of childhood cancer. Any patient with histologically proven Hodgkin lymphoma, not previously treated by chemotherapy, was included in this retrospective unicentric study, from 1 January 2005 to 31 December 2009. After a suspected case of Hodgkin lymphoma, a lymph node biopsy was performed by a pediatric surgeon, and the biopsy specimen was sent to Germany (Dr. Klaus Dunke, University of Münster) for pathology and immunohistochemistry. 

The diagnosis criteria for Hodgkin Lymphoma were those of WHO classification [7, 8].

Furthermore, all the patients had a full clinical examination, including otorhinolaryngologic examination and a number of additional tests such as blood count, sedimentation rate, renal and liver function tests, chest radiography, and abdominopelvic ultrasound. In view of our technical difficulties, some additional tests could not be done: EBV serology and CT scan nuclear medicine. Parental consent for treatment was obtained from parents or guardian of each child as part of treatment policy. 

Bone marrow biopsy has been performed in the two-stage III cases. Since our country does not have radiation therapy, our patients were treated exclusively by chemotherapy. The patients were treated with the hybrid COPP/ABV (Cyclophosphamide, Oncovin, Procarbazine, Prednisone, Adriamycin, Bleomycin, and Vinblastine) protocol used by the sub-Saharan pilot units of “Groupe Franco-African Paediatric Oncology” (GFAOP).

This chemotherapy was conducted as follows: vincristine 1.5 mg/m² day 1, cyclophosphamide 650 mg/m² day 1, procarbazine 100 mg/m² day 1 to day 7, adriamycine (Doxorubicin) 35 mg/m² day 8, vinblastine 6 mg/m² day 8, bleomycin 10 mg/m² day 8, and prednisone 40 mg/m² day 1 to day 14 (Table 1). 

Premedication with 25–50 mg of hydrocortisone hemisuccinate was administered IV slowly before each chemotherapy. The intertreatment interval was 28 days. 

The Ann Arbor classification was used for disease staging [9]. The absence or presence of fever higher than 38°C for 3 consecutive days, drenching night sweats, or unexplained loss of 10% or more of body weight in the 6 months preceding admission are to be denoted by the suffix letters A or B, respectively.

**Figure 1:**
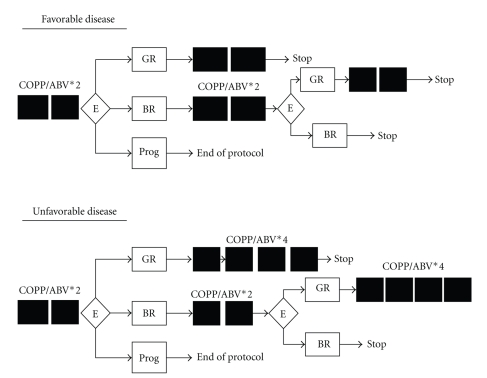
General treatment diagram. GR: good responder, BR: bad responder, E: evaluation, and Prog: progressive disease.

 Two treatment groups were defined in our protocol: favorable group (stages I and IIA, and mediastinothoracic index less than 0.45 and lymphadenopathy less than 6 cm and no contiguous involvement) and unfavorable group (stage IIB, III, IV or large mediastinum with mediastinothoracic index greater than or equal to 0.45, lymphadenopathy greater than or equal to 6 cm or visceral contiguity). For the favorable group, if an evaluation after two COPP/ABV courses showed a complete remission (disappearance of all clinical, biological, and radiological signs) or good response (disappearance of clinical and biological signs and reduction by at least 75% of all maximum diameter products of all measurable lesions), treatment was stopped after two additional courses. For the unfavorable group, in case of complete remission or good response after two courses, the treatment was stopped after four additional courses. On the other hand, for cases that do not respond well (no change or progress) after the first assessment to two courses, a second evaluation was made after two other courses, a good response after the four therapies led to four additional courses, and then the treatment was stopped. Progress of the disease entailed stoppage of the protocol and a palliative treatment (Figure 1). The data were analyzed on SPSS software, version 12. 

## 3. Results

During the study period, a total of 217 cases of childhood cancer were diagnosed and treated. Of these cases, 7 were Hodgkin lymphoma, which gives a hospital prevalence of 0.04%. Hodgkin lymphoma was the 7th most common cancer in our unit after Burkitt's lymphoma, retinoblastoma, nephroblastoma, acute leukemia, and malignant germ cell tumors. 6 out of 7 patients were from the city of Bamako. The mean age was 11.71 years, ranging from 6 to 15 years (Table 2). The sex ratio was 6 in favor of boys: 86% against 14%. 57.1% (4/7) of the fathers were farmers, 14.3% (1/7) were traders, and 71.4% (2/7) were civil servants. 85.7% (6/7) of the mothers were housewives and street vendors. 5 patients had a family of more than 7 members.

85.7% of the parents were of a disadvantaged socio-economic status. The mean time from the development of symptoms to diagnosis was 8 months. No diagnosis was made in less than 3 months. 2 cases were diagnosed after one year of evolution. At least one B sign was present in all the cases. Cervical lymphadenopathies were the signs found in 71.4% of the cases.

 71.4% (5/7) of the cases were stage IIB of Ann-Arbor classification and 28.6% (2/7) were stage IIIB (Table 3). All the patients belonged to the unfavorable group.

 4 out of 7 patients had lymph nodes whose size was between 2 cm and 4 cm, 2 patients had lymph nodes of 5 cm to 6 cm, and only one patient had lymph nodes of more than 7 cm. Fever was present in 3 out of 7 (42.8%). In two cases, there were night sweats. The three B signs (weight loss, fever, and night sweats) were present in two patients.

 In 6 of 7 cases (85.7%), the erythrocyte sedimentation rate was high (more than 10 mm at first hour); it was normal in one case. Histologically, the following were noted: 42.8% of scleronodular subtype, 28.6% of lymphocyte-rich subtype, 14.3% of mixed cellularity subtype, and 14.3% of lymphocyte-depleted subtype.

 3 patients had a hemoglobin level below 12 g/dl and 4 had a hemoglobin level between 12 and 14 g/dl. 3 patients (42.9%) had a widened mediastinum at diagnosis (>0.45 the thoracic diameter).

 34 courses of chemotherapy were evaluated: two deaths from drug toxicity were recorded.

 With a median followup of 37 months, 5 of 7 patients (71.4%) remain in complete remission, whereas 2 of 7 (28.6%) have died of complications of treatment.

## 4. Discussion

In 60 months, we recorded only 7 patients with Hodgkin lymphoma. The small sample size is due partly to the rarity of this disease and to the fact that people in other regions of Mali do not have access to our single national centre for cancer in children. People in the hinterland had difficulties accessing the centre due to poverty which affects the majority of Mali's population and the long distance.

 Although the diagnosis of cancer is generally difficult in most African countries due to insufficient resources and diagnostic facilities, it is recognized that Hodgkin lymphoma is rare in most of these countries, such as Nigeria where over 5 years Tanko et al. identified only three cases of Hodgkin's disease out of 87 (3.4%) pediatric solid cancers [5]. The same observation was made by Stiller, who admitted that Hodgkin lymphoma was relatively rare in many developing countries and Japan [10]. Makata et al. in Kenya also found that Hodgkin lymphoma accounted for only 4.1% of all pediatric solid cancers recorded in ten years from 1979 to 1994 [11]. Olweny et al. in Uganda treated in 10 years (1967–1977) only 48 patients under 16 years old with Hodgkin lymphoma [12]. In Dakar (Senegal), Ka et al. recorded only 7 cases of pediatric Hodgkin lymphoma in 11 years from January 1990 to December 2000 [13]. These results show that despite difficulties of diagnosis in Africa, Hodgkin lymphoma in children remains rare.

 Hodgkin lymphoma ranked seventh among cancers in children in our unit. Tanko in his series found that Hodgkin lymphoma was fifth among pediatric cancers [5].

 The average age in our series was 11.7 years with extremes of 6 and 15 years. As in other developing countries, the 6–14 years age group is most affected by Hodgkin lymphoma [13, 14].

Our average age is higher than that of Stefan et al. in South Africa, who found an average age of 8 years 11 months [15].

 In a multicentric study conducted in USA by Donaldson et al. on 110 children with Hodgkin lymphoma stage I and II, the average age was 13 years [16]. In another study conducted by Friedmann et al. in USA, 56 children were suffering from Hodgkin lymphoma (unfavorable group); the average age was 15 years [17].

 It appears that Hodgkin lymphoma is relatively rare in Blacks as shown in the same study by Donaldson, where only 11% of the patients were African-Americans as against 82% of Whites [16].

 It also appears that Hodgkin's disease is rare in developing countries where the peak incidence occurs before adolescence, as was the case in our study [9].

 In light of all these observations, we could say that the peak incidence of Hodgkin lymphoma is earlier in low income countries compared to that observed in high-income countries.

 In our series, male predominance is significant with a sex ratio of 6/1. The high sex ratio could be partly related to the small size of our sample. However, male predominance in Hodgkin lymphoma is reported by many authors in developed and developing countries [15, 17, 18].

 Histologically, the scleronodular subtype was dominant with 42.8% of the cases, followed by the lymphocyte-rich subtype (28.6%). The mixed cellularity subtype and lymphocyte depleted subtype had the same frequency of 14.3%. Engel in his South African series of paediatric Hodgkin lymphoma cases found 89% of the scleronodular subtype and the rest were mixed cellularity [18].

 In India, Laskar, with 251 Hodgkin lymphoma patients, found 71% of mixed cellularity subtype, 12% scleronodular subtype, 10% of predominantly lymphocytic subtype, and 7% of subtypes not specified [19].

 In Europe, from a multicenter study involving 47 centers, Noordijk et al. found, regardless of age, 75% of scleronodular subtype [20].

 Friedmann et al. in USA found, in a population of 56 children with Hodgkin lymphoma, 47 cases of scleronodular subtype, 6 cases of mixed cellularity, 2 cases of predominantly lymphocyte subtype, and one case that could not be classified [17].

 In Nicaragua, Baez and his team, with 48 patients admitted in 5 years, recorded 52.1% (25) cases of mixed cellularity, 31.2% (15) of scleronodular subtype, 10.4% (5) of lymphocyte-rich subtype, and 6.3% (3) of lymphocyte depleted subtype [21].

 In our study, and consistent with the literature, the scleronodular subtype is the most common [9].

 On the other hand, the mixed cellularity subtype is predominant in India and Latin America.

 71.4% of our patients had a B sign compared to Friedmann's study in which only 22 patients out of 56 (39%) had a B sign [17].

 Donaldson in USA found 5 cases of B sign out of 74 patients in stage II [16]. 

 As regards drug toxicity, we observed two toxic deaths by febrile neutropenia. These deaths occurred despite an empirical triple antibiotic treatment. Difficulties in obtaining additional laboratory examinations to document these infections and adapt the antibiotic treatment and the unavailability of blood culture or supportive growth factors could be the cause of these deaths. The COPP/ABV hybrid protocol seemed to be well tolerated in all our patients. This ABVD combination was recognized as being of low toxicity. Introduced in the 1970s by Bonadonna and his Italian team into the therapeutic arsenal of Hodgkin lymphoma, it is now considered the gold standard treatment for this disease. In addition, it has less sterile and less leukemogenic activity [3, 22].

 Shimabukuro-Vornhagen and his team in Germany treated 2,715 patients with Hodgkin aged 16–71 years with COPP/ABVD (COPP/ABV plus Doxorubicin) for the early stages and BEACOPP (Bleomycin, Etoposide, Doxorubicin, Cyclophosphamide, Vincristine, Procarbazine, and Prednisone) for the advanced stages. They recorded only three toxic deaths [23]. 

 About nonlethal toxicity, we recorded one case of alopecia, 2 cases of digestive toxicity (nausea and vomiting), and one case of nonfebrile neutropenia. These findings were in agreement with those described in the literature [24].

 With a median followup of 37 months, our overall survival rate is 71%. This rate is significantly higher than that obtained by the Senegalese who, over a period of 11 years, treated seven patients, with six deaths and one lost to followup. Our result is comparable to that obtained by Olweny in Uganda who used virtually the same protocol like us (75% for stages I–IIA and 60% for stages IIIB and IV), but our sample is much smaller [12]. 

 The 95% overall survival rate obtained by teams in developed countries is not comparable to our poor results [3].

 We are not comparable to the 95.7% overall survival rate of localized stages obtained by the French Pediatric Oncology Society [25].

 Hodgkin lymphoma remains relatively rare among African children, but multicentric studies admitting a larger number of young patients with this condition are needed to further fine-tune the results.

## Figures and Tables

**Table 1:** GFAOP Hodgkin Lymphoma treatment Protocol.

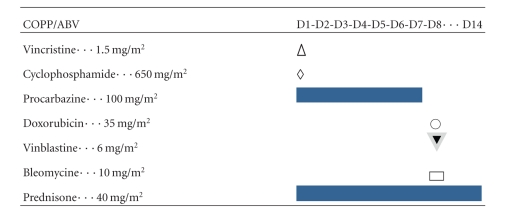

D: day.

**Table 2:** Characteristics of patients.

Characteristics	Number	Percentage
Sex		
Male	6	85.7
Female	1	14.3
Age		
Median	12 years	
Histology		
Scleronodular	3	42.8
Lymphocyte-rich	2	28.6
Mixte cellularity	1	14.3
Lymphocyte depleted	1	14.3
Stage		
IIB	5	71.4
IIIB	2	28
Fever	3	42.8
Weight loss	2	28.6
Night Sweats	2	28.6
Weight loss + fever	2	28.6
Weight loss + fever + night sweats	2	28.6
High sedimentation Rate	6	85.7
Diagnosis delay		
<3 months	0	0
4–12 months	5	71.4
>12 months	2	28.6
Hemoglobin < 12 g/dl	3	42.8
12 ≤ Hemoglobin ≤ 14	4	57.2

**Table 3:** Additional patient's characteristics and treatment results.

Sex	Patient 1	Patient 2	Patient 3	Patient 4	Patient 5	Patient 6	Patient 7
	Female	Male	Male	Male	Male	Male	Male
Time to diagnosis	4–12 months	4–12 months	4–12 months	4–12 months	4–12 months	>12 months	>12 months
Age	10 years	8 years	15 years	6 years	13 years	15 years	15 years
Stage	II	II	II	II	II	III	III
Histology	LR	LR	SN	SN	SN	MC	LD
Treatment	6 C	6 C	6 C	6 C	6 C	2 C	2 C
Response	CR	CR	CR	CR	CR	D	D
Progression-free survival	57 + months	47 + months	30 + months	29 + months	20 + months	D	D

C: courses, CR: complete remission, D: deceased, LR: lymphocyte-rich, SN: scleronodular, MC: mixte cellularity, LD: lymphocyte-depleted.
